# Supplementary material for: Finding help and hope in a peer-led reentry service hub near a detention centre: A process evaluation
Source: PLoS One. 2023 Feb 17;18(2):e0281760. doi: 10.1371/journal.pone.0281760 (PMC9937468; doi:10.1371/journal.pone.0281760)
Supplement: S1 File — (PDF) [file pone.0281760.s001.pdf]

Reintegration Centre – Needs Identification Tool

**Name** (First/Last):	**Date of Birth** (dd/mm/yy):<br>/ /
**Health Card Number:**	
**Telephone/Cell:**	**Email:**
**Address:**	
**Nearest Major Intersection:**	**Gender:** Male ☐ Female ☐ Trans ☐

Is this your first time in custody? Yes ☐ No ☐
How long were you in custody? ________
When you were in custody were you: Sentenced ☐ On Remand ☐ Not discussed ☐
Reason for current justice involvement: New charge ☐ Breach ☐ Not discussed ☐
Does client have any charges / convictions that may affect service delivery? No ☐ Yes ☐

**Sexual Identity:**	Heterosexual ☐ Lesbian ☐ Gay ☐ Bisexual ☐ Queer ☐ Questioning ☐ Not disclosed ☐ Other: _____________________________
**Preferred Language(s) for Receiving Services:**	
**Canadian Residency Status:**	Citizen ☐ Permanent Resident ☐ Protected Persons ☐<br>Non-status Person ☐ Temporary Worker ☐ Prefer Not to Disclose ☐<br>Other: _________________________________<br>Arrival in Canada (yyyy):
**Racial and/or Ethnic Identity:**	☐ Asian<br>☐ Asian – East (i.e. Japanese, Chinese, Korean)<br>☐ Asian – South (i.e. Indian, Pakistani, Sri Lankan)<br>☐ Asian – Southeast (i.e. Malaysian, Pilipino, Vietnamese)<br>☐ Black<br>☐ Black – African (i.e. Ghanaian, Kenyan, Somali)<br>☐ Black – North American (i.e. Canadian, American)<br>☐ Black – Caribbean (i.e. Barbadian, Jamaican)<br>☐ First Nations<br>☐ Indigenous/Aboriginal not included elsewhere<br>☐ Inuit<br>☐ Metis<br>☐ Francophone<br>☐ Latin American (i.e. Argentinian, Chilean, Salvadoran)<br>☐ Middle Eastern (i.e. Egyptian, Iranian, Lebanese)<br>☐ West Indian (i.e. Guyanese with origins in India)

	☐ White<br>☐ White – European (i.e. English, Italian, Portuguese, Russian, Ukrainian)<br>☐ White – North American (i.e. Canadian, American)<br>☐ Mixed Heritage (i.e. Black-African and White-North American) (Please specify: ______________________________________)<br>☐ Other (Please specify: _______________)<br>☐ Prefer Not to Answer<br>☐ Do Not Know

**Social support:**<br><br>When things go badly, who do you turn to? (check all that apply)	☐ Friends
	☐ Spouse/partner
	☐ Parents
	☐ Siblings
	☐ Other relatives
	☐ Work buddies
	☐ Service Provider
	☐ Clergyperson/Religious leader
	☐ Adult Children
	☐ Nobody
	☐ Other (specify) __________________
	☐ Not Discussed

Children:

Do you have any children? Yes ☐ No ☐ Not discussed ☐
If so, how many? _____

Child:	Age:	Are you in contact with them?
#1		Yes ☐ No ☐
#2		Yes ☐ No ☐
#3		Yes ☐ No ☐
#4		Yes ☐ No ☐
#5		Yes ☐ No ☐
#6		Yes ☐ No ☐
#7		Yes ☐ No ☐

Please list services that you are currently accessing (i.e. mental health, medical, housing):

**Organization:**		**Contact Person:**	
**Telephone:**	**Ext.**	**Fax:**	**Email:**
**Program or Service:**		**Reason for service use:**	

**Organization:**		**Contact Person:**	
**Telephone:**	**Ext.**	**Fax:**	**Email:**
**Program or Service:**		**Reason for service use:**	

**Organization:**		**Contact Person:**	
**Telephone:**	**Ext.**	**Fax:**	**Email:**
**Program or Service:**		**Reason for service use:**	

**Organization:**		**Contact Person:**	
**Telephone:**	**Ext.**	**Fax:**	**Email:**
**Program or Service:**		**Reason for service use:**	

**Health:**	In general, would you say your <u>mental</u> health is:<br><br>1) excellent? ☐ 2) very good? ☐ 3) good? ☐ 4) fair? ☐ 5) poor? ☐
	In general, would you say your <u>physical</u> health is:<br><br>1) excellent? ☐ 2) very good? ☐ 3) good? ☐ 4) fair? ☐ 5) poor? ☐
	Do you have a family doctor that you see most of the time?<br>Yes ☐ No ☐ Not discussed ☐<br><br>If you don’t have a family doctor, where do you go when you need health care?

**Traumatic Brain Injury:**	Have you ever had a head or neck injury that knocked you out or at least left you dazed, confused, or disoriented? Yes ☐ No ☐ Not discussed ☐
	How many injuries like this have you had over your lifetime? ______

**Trauma:**	I'd like to ask you about some past events that might have been difficult for you.<br><br>Have you ever been in a serious accident?<br>Yes ☐ No ☐ Not discussed ☐<br><br>Have you ever been in an environmental or ecological disaster such a fire, tornado, hurricane, flood, earthquake, or chemical spill?<br>Yes ☐ No ☐ Not discussed ☐<br><br>Have you ever been in any situation in which you were seriously injured or in a situation in which you feared you might be seriously injured/killed?<br>Yes ☐ No ☐ Not discussed ☐<br><br>Have you ever witnessed a situation in which someone was seriously injured/killed, or have you ever witnessed a situation in which you feared someone would be seriously injured/killed?<br>Yes ☐ No ☐ Not discussed ☐

**Please indicate the areas in which the individual has an immediate need:**	**Required**	**Not Needed**	**Not Discussed**	**Strength**
1. Housing<br>a) Immediate Housing/Shelter<br>b) Short-term Housing<br>c) Long-term Housing<br><br>Comments:	 ☐<br>☐<br>☐	 ☐<br>☐<br>☐	 ☐<br>☐<br>☐	 ☐<br>☐<br>☐
2. Food<br><br>Comments:	☐	☐	☐	☐
3. Clothing<br><br>Comments:	☐	☐	☐	☐
4. Transportation<br><br>Comments:	☐	☐	☐	☐
5. Income Benefits<br>a) Ontario Works<br>b) Ontario Disability Support Program<br><br>Comments:	 ☐<br>☐	 ☐<br>☐	 ☐<br>☐	 ☐<br>☐
6. Identification Documents (i.e. driver's license, OHIP)<br><br>Comments:	☐	☐	☐	☐
7. Healthcare<br>a) Medication<br>b) Primary Physician<br>c) Dental<br>d) Vision<br>e) Hearing<br>f) HIV/HEP-C<br>g) Acquired Brain Injury (ABI) Supports<br>h) Fetal Alcohol Spectrum Disorder (FASD) Supports<br>i) Developmental Disability	 ☐<br>☐<br>☐<br>☐<br>☐<br>☐<br>☐<br>☐<br>☐	 ☐<br>☐<br>☐<br>☐<br>☐<br>☐<br>☐<br>☐<br>☐	 ☐<br>☐<br>☐<br>☐<br>☐<br>☐<br>☐<br>☐<br>☐	 ☐<br>☐<br>☐<br>☐<br>☐<br>☐<br>☐<br>☐<br>☐

Please indicate the areas in which the individual has an immediate need:	Required	Not Needed	Not Discussed	Strength
j) Physical Needs<br><br>Comments:	☐	☐	☐	☐
8. Legal Assistance<br>a) Legal Aid<br>b) Immigration Issues<br>c) Custody Issues<br><br>Comments:	☐<br>☐<br>☐	☐<br>☐<br>☐	☐<br>☐<br>☐	☐<br>☐<br>☐
9. Mental Health and Substance Use Supports<br>a) Mental health supports<br>b) Substance use supports<br>c) Harm Reduction / Outreach<br>d) Overdose prevention<br>e) Concurrent disorder<br>f) Dual diagnosis<br><br>Comments:	☐<br>☐<br>☐<br>☐<br>☐<br>☐	☐<br>☐<br>☐<br>☐<br>☐<br>☐	☐<br>☐<br>☐<br>☐<br>☐<br>☐	☐<br>☐<br>☐<br>☐<br>☐<br>☐
10. Social Supports<br>a) Culture Supports<br>b) Language/Translation Supports<br>c) LGBTQ Supports<br>d) Trauma Supports<br>e) Childcare<br>f) Family Access/Custody<br>g) Peers Supports<br>h) Spirituality<br><br>Comments:	☐<br>☐<br>☐<br>☐<br>☐<br>☐<br>☐<br>☐	☐<br>☐<br>☐<br>☐<br>☐<br>☐<br>☐<br>☐	☐<br>☐<br>☐<br>☐<br>☐<br>☐<br>☐<br>☐	☐<br>☐<br>☐<br>☐<br>☐<br>☐<br>☐<br>☐
11. Education and Training<br>a) Skills training<br>b) Literacy skills<br>c) Life skills<br>d) High School diploma (GED)<br>e) Pre-employment support<br>f) Computer training	☐<br>☐<br>☐<br>☐<br>☐<br>☐	☐<br>☐<br>☐<br>☐<br>☐<br>☐	☐<br>☐<br>☐<br>☐<br>☐<br>☐	☐<br>☐<br>☐<br>☐<br>☐<br>☐

Please indicate the areas in which the individual has an immediate need:	Required	Not Needed	Not Discussed	Strength
g) Self-employment support<br><br>Comments:	☐	☐	☐	☐
12. Other needs<br><br>Comments:	☐	☐	☐	☐

Client Priorities

Please list *the client's* top 3 priorities in meeting the needs identified on the previous page:

First Priority:	
Second Priority:	
Third Priority:	
Was there one priority / area that client spent the most time talking about? If so, please indicate:	

Client Strengths

Please identify *the client's* strengths:

**Resiliency:**	Please rate how much this statement applies to you:<br><br>I tend to bounce back quickly after hard times.<br>☐ Strongly agree<br>☐ Agree<br>☐ Neither agree nor disagree<br>☐ Disagree<br>☐ Strongly disagree<br>☐ Not discussed

Referral Information

**Lead Service Provider:**			
Lead Service Provider Contact:			
Telephone:	Ext.:	Fax:	Email:
Referral Completed: Yes ☐ No ☐	Date of Completed Referral:		
Client Starts Service: Yes ☐ No ☐	Date Service Begins:		

**Second Service Provider:**			
Second Service Provider Contact:			
Telephone:	Ext.:	Fax:	Email:
Referral Completed: Yes ☐ No ☐	Date of Completed Referral:		
Client Starts Service: Yes ☐ No ☐	Date Service Begins:		

**Third Service Provider:**			
Third Service Provider Contact:			
Telephone:	Ext.:	Fax:	Email:
Referral Completed: Yes ☐ No ☐	Date of Completed Referral:		
Client Starts Service: Yes ☐ No ☐	Date Service Begins:		

How did you hear about the RC?

____
____
____

Comments (Agreed to by Client and Worker)

____
____
____
____

**Name of Person Completing this Form:**			
Name of Organization/Program:			
Date of Completion (dd/mm/yy): / /			
Telephone:	Ext.:	Fax:	Email:
Time in:	Time out:		
