# Supplementary material for: Finding help and hope in a peer-led reentry service hub near a detention centre: A process evaluation
Source: PLoS One. 2023 Feb 17;18(2):e0281760. doi: 10.1371/journal.pone.0281760 (PMC9937468; doi:10.1371/journal.pone.0281760)
Supplement: S2 File — (PDF) [file pone.0281760.s002.pdf]

Appendix 6

John Howard Poverty Reduction Project
Client Interview Guide (Group A_Subset)

Interviewer's Name:
Participant ID:
Date:
Interview Location:

INTRODUCTION

You have been invited to share your experiences since we met at the Reintegration Centre. Thank you for agreeing to participate in this interview. I mentioned in the consent letter that the interview should take about an hour. We do appreciate the time you're giving us today and don't want to keep you here longer than an hour so we'll do what we can to stay within the time limit.

[Start Audio Recorder] This is [interviewer's name] and it is [date] and I am here with [participant ID] at [interview location] and we are about to start the interview.

I'm going to read each question for you and at times will ask you to provide more detail for some of the questions or ask additional questions based on your responses. Please let me know at any time if the meaning of the question is unclear and I will ask it in another way. I'd like you to answer in your own words. We are interested in your experiences when responding to the questions. Please remember, there are no "right or wrong" answers. We are really interested in your experiences so all of the information you provide is important.

Topic**: Client Experiences of Re-integration & Resiliency**

1. **As we start, I'd like to know how things are going for you right now.**
2. **Has anything changed since we last met a month ago?**

3. **I'll start by asking you to describe what it was like to be back in the community after your last release from TSDC?**

4. **How have you been feeling during the last month?**

   Probes:
   - What kind of challenges did you experience during your last release from [TSDC]?
   - What went well for you during your last release from [TSDC]?
   - How did you spend those days?
   - Where did you stay? Who did you spend time with?

5. **Can you tell me whether you felt 'ready' to return to the community/to leave TSDC?**

   Probes:
   - How did you feel unprepared/prepared?
   - What would have made you feel better prepared?

Topic**: Client Priorities**

---

6. **When we last spoke at the RC you talked about your priorities. I know it was a while ago now, but at the time you mentioned working towards [priority 1-3]**

   Probes:
   - Have you had time to work on any of these priorities?
   - How's it going?
   - [probe specifics on the priorities identified]

7. Were you referred to someone who could help you with your priorities?

Probes:

- Can you take me through that experience?
- What about it was helpful or not helpful?

Topic**: Housing**

---

8. Can you talk about your living situation in the last month?

Probes:

- Where have you stayed in the last month?
- Who did you live with?
- Where did you live?
- Have you ever found yourself without a place to sleep?
  - Can you tell what that was like?
- Who did you turn to for help?

Topic**: Health & Illness**

---

9. I'd like to ask you a few questions about your health. How has your health been over the last month? What health issues have you had to deal with since your release?

Probes:

- Have you had any physical health problems?
- Have you had any other health problems?
- Did you get help for this/these problems?
  - If no, what blocked you from getting help?
  - If yes, how long did it take for you to get help?
    - Who helped?
  - Where did you go to get help?
  - Did you face any difficulties to getting help?

Topic**: Support System**

---

10. Who do you turn to when you have a problem?

Probe:

- Can you take me through the last major problem you had?

11. In this situation, who did you turn to?

12. What are some of your favorite places to go in the community?

Probe:

- If service setting, ask about the service/provider

13. Who do you turn to in the community/neighbourhood when you need help?

Probes:

- In what ways are they supportive or how do they help you/show you they care?
- Is there anyone else in your community that helps you?
- What types of help have you tried to get in the community (for example, housing support)?
  - In what way were they helpful to you?
  - How were they not helpful?

14. Was there ever a time when you reached out or needed help and didn’t get it?

Probe:

- Can you take me through this?

Topic**: Coping**

---

15. You told me about some of the challenges you have faced since your release. I would like to know how you deal (or cope) with these kinds of challenges?

Topic**: Family & Parenting** (reminder – safe space and confidential)

I'd like to learn more about your relationship with your family and children.

16. Who, in your family, do you have contact with?

Probes:
- We all have different definitions of family, so when I say family, who comes to mind?
- How often do you see (them, those identified above)?

17. (If they haven't mentioned children) Do you have any children?

Probes: (If yes,)
- How often do you see them?
- How would you describe your relationship with your children?
- Was there a time when your relationship was different?
  - What caused it to change?
  - Would you like it to be different?
  - How would you like it to be different?

Topic**: Relapse & Harm Reduction** (Reminder – safe space and confidential)

I'd like to ask you about drug and/or alcohol use. We are aware that we're currently in a fentanyl crisis.

18. Since returning to community have you used drugs or alcohol?

Probes:

- What led you to use again?
- Have you tried to not use/drink since you returned?
- What has gotten in your way of this?
- Has anyone helped you to stop/change your drug/alcohol use?

19. Did you receive any help from the RC on how to protect yourself when using?

Probes:

- What information or supplies were helpful?
- What could they have done to better help you?

Topic**: Finances and Employment** (Reminder – safe space and confidential)

I'd like to learn more about what you are doing with your time now that you are back in the community.

20. How have you supported yourself financially since you release?

Probes:
- Do you have enough money to get by?
- What kind of things do you do to get by?
- Can you tell me more about that?

21. Have you been able to work since your release?

22. Can you tell me a bit about your experiences looking for work in recent months?

Probes:
- What kind of obstacles have you faced?
- Did you seek out any help with this?
  - If yes, from where?
  - How did that go?
- What kind of help did you need, but didn't get?

23. Have you ever been let go from a job? (anytime, not in the past month)

Probes:
- Can you tell me what happened?
- What has been difficult about keeping a job?

Topic**: Education/training**

I'd like to know about your educational background and training.

24. Did you train for or go to school for a specific job/career?

25. Do you have any plans to upgrade your skills or go back to school?

Probes:
- Can you tell me more about this?
- How are you working towards this?
- Is there something else you are focusing on besides upgrading skills or going back to school?

Topic**: Goals**

---

26. Can you tell me a bit about the goals that you set for yourself after release?

27. Where would you like to see yourself in a year or two from now? Can you describe that for me?

28. How do you think you can stay on track with that/these goal(s)?

29. What type of help from John Howard Society would you need to stay on track with these goals?

30. What type of help would you need to avoid being back where you were several months ago?

Turn Over

Closing statements/questions:

We talked about many things today. Thank you for sharing this with me.

As this comes to an end, is there anything you would like to add? Is there something I didn't ask but should have about your experiences following release? Is there anything else you would like to say about your experience of returning to the community?
