# Supplementary material for: Finding help and hope in a peer-led reentry service hub near a detention centre: A process evaluation
Source: PLoS One. 2023 Feb 17;18(2):e0281760. doi: 10.1371/journal.pone.0281760 (PMC9937468; doi:10.1371/journal.pone.0281760)
Supplement: Attachment — Submitted filename: Response to the Reviewer-V1_Final.docx [file pone.0281760.s004.docx]

Reviewer #2:

Comment 1: After reading through the revised version of this manuscript, I appreciate several of the additions that the authors have made in an attempt to improve it. The discussions of prior qualitative research on reentry and desistance experiences generally helped to contextualize the current work more, and more information on the nature of qualitative process evaluations was helpful as well. Overall, I think that the manuscript is much-improved, but there are still a couple of lingering issues.

*Response 1: Thank you.*

Comment 2: The in-text description of the JHS’s intake needs assessment (and its inclusion as an appendix) was also helpful, but for me this raises more questions that are unanswered in the current iteration of the manuscript. This study was intended to be a process evaluation, and a large part of the “process” of the Reintegration Centre revolves around referring clients to services on the basis of that needs assessment. The importance of “individualized service referrals” is mentioned specifically in the Abstract and elsewhere in the manuscript. However, the assessment does not appear to be “scored” in any actuarial sense, and it is not clear how peer support workers are trained to conduct this assessment with any sort of rigor. Can the authors provide any insight on that training and how support workers identify their clients’ greatest needs? Are there any ways that this assessment could be improved? Should the Centre adopt a validated, actuarial assessment tool like the LSI-R or otherwise in order to better support services in the areas clients need it the most?

Response 2:

The reviewer acknowledges that the details we added on JHS intake needs assessment were helpful but raised additional questions.

Before responding to these concerns as carefully and comprehensively as we can, we preface what follows with what we see as the broader issue from which these concerns seem to spring.

That is, the thrust of the concerns hinge on a key distinction between JHS’s needs assessment as an interpretive, interactive, emergent process, on the one hand, and the RC intake form as an instrument that peers use to facilitate and inform that broader process (given the interactional affordances the object offers in situ). We delineate the distinction as well as the risks of conflation below, as we attempt to respond to the interrelated set of concerns raised by the reviewer.

We would emphasize that “referring clients to services” is not solely, or even mostly, “***on the basis of*** that needs assessment [instrument—the intake form],” but rather on the basis of a broader interpretive and interactive process of assessment, comprising multiple, interrelated dimensions and subprocesses, including, for example:

*Developing rapport*

*Establishing trust*

*Managing reservations*

*Engaging in sympathetic introspection*

*Identifying, defining, describing, and discussing strengths*

*Identifying, defining, describing, and discussing challenges and obstacles*

*Identifying, defining, describing, and discussing challenges and (service) needs*

*Taking stock of habits, dispositions, tendencies, preferences*

*Identifying reentry priorities, establishing reentry goals, and making reentry plans*

*Considering options and alternatives*

From this view, making effective referrals to services is contingent on not only developing a shared understanding (i.e., intersubjectivity) of the salient strengths, obstacles, opportunities, needs, and priorities at reentry, but also on establishing a social space and relational style that invites and fosters peer-client co-empowerment, with each exercising agency in shaping the referral process as it unfolds.

The responses to the intake form in the field were not scored “in any actuarial sense,” as the reviewer rightly notes, but this was by design. The context of clients heretofore is one in which their status as persons, their selves, their identities have been defined in significant ways by the metrics of imprisonment, risk assessment, discharge planning, and community reentry. When prospective clients arrived at the RC, they were coming from a context in which they continually confronted the ritual degradation of self via the people-processing institutions of the criminal justice system, often through ostensibly valid, objective instruments of quantitative assessment. Asking them to submit to yet another actuarial assessment may further marginalize prospective clients at the critical moment of release from custody, sabotaging peer-client rapport, and prompting refusals of referral.

The metricization of marginalization and the construction of risky subjects, to be sure, is also a feature of the program evaluation research literature more generally. And notwithstanding the importance of quantitative assessment, there are also risks associated with relying on quantitative instruments of assessment, such as reducing and oversimplifying complex processes to static indicators as well as reducing both the face and ecological validity of referrals. In short, there are tradeoffs associated with every approach, relative to others, one needs to ask what’s won? What’s lost? What’s a wash?

*While the LSI-R is designed to identify people’s risks and needs with regard to recidivism, including their particular criminogenic needs, the goal of the John Howard Reintegration Center’s assessment tool was intended to facilitate a conversation around clients’ immediate needs to ensure appropriate referral to services and supports While the LSI-R is a widely used tool within the correctional system, we would not advise implementing the LSI-R in community based agencies that are designed to support and empower people, particularly at the moment of release. Notwithstanding that some have noted that risk/needs tools can be supplemented with more strengths-based assessment tools that empower, promote well-being, and have additional benefits such as providing invaluable case planning information [3, 4], the concern would be that the community based agencies would be seen as an arm of the correctional system, which and likely inhibit and erode any sense of security for clients..*

Given the relational, strengths-based approach to assessment as a process to which the John Howard Reintegration Centre subscribed, issues such as training peer workers “to conduct this assessment with any sort of rigor” become less consequential given this more comprehensive view of assessment as a complex, contingent, patterned, yet not-fully-predictable process.

With respect to the training associated with the intake form, the formal training period of the peers predated the project, both in terms of its design and course. There’s also an important distinction between training and doing/implementing in practice, that we would highlight and emphasize. That is, although training would be within the scope of a process evaluation, much more consequential for our purposes is what people actually do in practice, including the formal and informal processes of “learning the ropes” along the way. And thus while we do provide some insight into the pedagogical approach to the formal training associated with the intake form, doing so risks misrepresenting the form, inflating its importance in the broader relational, emergent, interactive, interpretive process of needs assessment to which John Howard Society Toronto and the peers subscribed.

Relatedly, “[H]ow support workers identify their clients greatest needs” is multidimensional interpretive and interactive process, beginning with developing rapport, establishing trust, introducing the instrument, defining competencies and dispositions, puzzling through problems, pursuing solutions, clarifying concerns, engaging in ongoing reflection on opportunities and obstacles given current circumstances, and so on. As a subject of evaluation, training as an ongoing process, while analytically interesting and practically consequential, is beyond the focus and scope of this paper. We would, however, encourage future work on peer training as a social process.

***The intake form was developed by/with the Centre for Addiction and Mental Health (CAMH: Toronto, Ontario) and the John Howard Reintegration Centre – and prior to the development of the research project – with several goals, including peer and client empowerment through questions that center on client well-being and strengths. With respect to the training and identification of client needs it is our understanding that, at the time of development, CAMH staff provided the peer support workers with training that would support the peers to complete the intake form using the principles of Motivational Interviewing, empowering the peer support workers with the freedom to complete the tool in a conversational manner. In essence the form is a 2-way empowerment tool for peer support workers and clients, moving away from deficit based models that dominate the field.*** *In our opinion a strengths-based approach is essential to support people who have experienced the stigma of criminalization [1, 2].  – NOTE: text in green font is included in the revised manuscript.*

Please note that we did not include additional information regarding this comment into the paper at this time. If the reviewer and editor feel some additional

Comment 3: In my review of the initial submission, I made note of the fact that only 21 people participated in this study out of an initial 209 who were approached. The authors have added a few sentences to the Discussion section noting this and the difficulties of recruitment, but these additions do not address the crux of the issue that I mentioned: People who choose not to participate in a study tend to differ in many ways from those who do participate. It is entirely possible that a large portion of people who chose not to participate in this study did not find the program to be useful, or that they differ in some other aspect germane to the findings of the study. This limitation should be noted substantively in the Discussion.

Response 3: This limitation has been added to the paper as noted below.

“We do not know why 188 people approached to participate in the qualitative interview declined; they may differ in substantive ways from participants.”

1.	Feingold ZR. The stigma of incarceration experience: A systematic review. Psychology, Public Policy, and Law. 2021;27(4):550.

2.	Tyler ET, Brockmann B. Returning Home: Incarceration, Reentry, Stigma and the Perpetuation of Racial and Socioeconomic Health Inequity. Journal of Law, Medicine & Ethics. 2017;45(4):545-57. doi: 10.1177/1073110517750595.

3.	Fedock G, Covington SS. Treatment of Incarcerated Women and Girls. 2022.

4.	Wanamaker KA, Jones NJ, Brown SL. Strengths-based assessments for use with forensic populations: A critical review. International Journal of Forensic Mental Health. 2018;17(2):202-21.
