# Supplementary material for: Finding help and hope in a peer-led reentry service hub near a detention centre: A process evaluation
Source: PLoS One. 2023 Feb 17;18(2):e0281760. doi: 10.1371/journal.pone.0281760 (PMC9937468; doi:10.1371/journal.pone.0281760)
Supplement: Attachment — Submitted filename: Response to Reviewer 3_FINAL.docx [file pone.0281760.s005.docx]

Response to Reviewer #3

COMMENT 1: The sentence added in the limitations section ("We do not know why...") reads like it needs some context, I would encourage the authors to revise it.

RESPONSE: Good catch. The statement was added as per a previous reviewer’s suggestion. We agree, however, that it could be better phrased and contextualized. Thank you for the opportunity to do so. We have revised discussion of potential limitations as follows:

We also acknowledge the limitations of this process evaluation. First, considering the complexity of the needs among this population, and that the interviews were conducted within a short time after they were released from jail, the findings reflect a snapshot of their experiences in the early days after release and therefore do not offer a window into longer-term circumstances, relationships, processes, or outcomes. Still, research indicates that the first month after release is characterized by high health risks and poor health outcomes, such as high rates of overdose and mortality [35-38], which not only underscores the critical need for rapid supports, service connections, and continuity of care, but also warrants shorter interview follow-up periods when conducting health-informed reentry program evaluation research [35, 37, 115, 116]. Participants emphasized that the support they received from the Reintegration Centre was a critical factor in helping them navigate the high-risk period immediately following release and begin addressing health concerns, participating in their local communities, and rebuilding their lives. These findings are consistent with what other studies have found: community-based, post-release support services and interventions play a critical and positive role in enhancing community reintegration and reducing the risk of reincarceration [23, 117, 118].

While the results shed light on the early reentry experiences of those participating in a novel intervention, another potential limitation of the study is that the findings draw on qualitative interviews with 21 men, a subset of the 209 individuals who were invited to participate in the research, which may have affected the range of perspectives we could solicit in this evaluation. The experiences and perspectives of interview participants may differ in substantive ways from others. More generally, we would also emphasize that the challenges of reentry--a situation in which individuals are juggling competing priorities and complex needs at a transitional time wrought with stress and hardship[119]--can also act as barriers to research participation [93]. For example, with respect to maintaining participant contact and scheduling interviews, the study of people facing personal, social and health challenges post-release often poses additional recruitment and retention challenges than researchers might encounter in general population or treatment samples. We experienced and adjusted to these challenges throughout the study, but there were 28 people who agreed to an interview, but later could not be reached for scheduling.

COMMENT 2: Under findings, page 23, the paragraph in the middle of the page "Interactions with social service providers..." needs a quote to support it.

RESPONSE: Thank you for the suggestion. In addition to the original citation to extant research supporting the statement (#100), we added illustrative material from our study data, as the reviewer suggested. The paragraph now reads as follows:

Interactions with social service providers can often be frustrating, even humiliating, especially for people experiencing complex needs [100]. For example, when asked if there were times “when you reached out or needed help and you didn't get it?” one participant lamented, *“All the time. That's regular. Nobody wants to help. Most people just want to act like… ‘Sit there and listen’-- and then tell you you're the problem, everything is your fault!”* (P12). Others expressed similar frustrations. For instance,

*Well the [reentry] challenges are just trying to get everything done and nobody's helping me so I just, like I said, I just put my hat, panhandle and make my money to get by … I'm trying to get my income together and everything else just screwing me up … You know, I make an appointment … for the clothing bank and the old lady was a bitch with me on the phone … She wasn't paying attention. Oh, this is, rah, rah, rah, rah so yeah. I'm stressed out … [the disability support program] won't give me any money. Welfare won't give me any money. I have an appointment coming up [with another service provider] for housing, so if I don't have the income, I got no money. I got no income and they can't do anything for me so I'd lose my subsidized housing. It's just been rough [returning to the community] … All I do is put out my hat every day and panhandle and so I have enough money to live off of … and also running around trying to get my [disability support application] together, trying to do Welfare, trying to do whatever I can getting it together so I can get a life*. (P13)

Similarly, after being informed that he might be in violation of his probation order -- almost immediately upon release -- for accessing the services of the Hub to arrange access to drug treatment, rather than reporting directly to a drug treatment program, another participant shared:

*[I was told] ‘You might get charged for a breach, you know.’ I had enough, like when you have that pressure, it makes you want to go use … These people are … not even helping you and probation is supposed to help people, like you’re on probation, but they’re supposed to tell you, guide you, help you, and get you help. It seems like they don’t do that.* (P05)

The reviewer and readers will also see that we provide and discuss additional examples of challenging, frustrating interactions in accessing services later on in the results—set in comparative context vis-à-vis Hub peer worker service encounters—in “Becoming a trusted reentry resource,” including, for example, the following responses from P07:

*“I've used this as my main backbone, like this is my rock because no other agency in the last month has given me as much help as this place. I mean, that interview we had when I first got out, we sat for what two, three hours? No other agency would even give me the time of day. They wouldn't even give me a half hour …. every question that I’ve had, every venture that I want to try, every advice that I need, it’s been answered. And it’s very rare for me to find a program that does that.*” (P07)

*[At other organizations] I go to meet a worker, and you can tell that they don’t have the life experience because they’re just sitting there like a deer in headlights. No, you don’t because you haven’t walked a second in my shoes. You have, K [peer support worker – name withheld]. You have. That’s why I like John Howard.”* (P07)

*“I have severe trust issues. I don't want to be going to a million different workers and having to re-explain everything over and over and over again because I’ve had to do that for ten fucking years.... I don’t want to have to re-explain my situation to worker after worker.”* (P07)

COMMENT 3: There are different font sizes in the paper (introduction vs findings).

RESPONSE: Thank you. This has been corrected.

COMMENT 4: I would recommend that the authors proof-read the paper carefully as there are some minor editorial issues - e.g. line 303 - "One coder [AM] on those that addressed".

RESPONSE: Thank you for pointing out this typo, which we’ve addressed in the current revision. We also did a full proof of the paper and tracked changes.

1.	Binswanger, I.A., et al., *Release from prison--a high risk of death for former inmates.* N Engl J Med, 2007. **356**(2): p. 157-65.

2.	Kouyoumdjian, F.G., et al., *The health care utilization of people in prison and after prison release: A population-based cohort study in Ontario, Canada.* PLoS One, 2018. **13**(8): p. e0201592.

3.	Zlodre, J. and S. Fazel, *All-cause and external mortality in released prisoners: systematic review and meta-analysis.* Am J Public Health, 2012. **102**(12): p. e67-75.

4.	Kouyoumdjian, F., et al., *Health status of prisoners in Canada: Narrative review.* Can Fam Physician, 2016. **62**(3): p. 215-22.

5.	Stewart, L.M., et al., *Risk of death in prisoners after release from jail.* Australian and New Zealand Journal of Public Health, 2004. **28**: p. 32-36.

6.	Verger, P., et al., *High mortality rates among inmates during the year following their discharge from a French prison.* Journal of Forensic Sciences, 2003. **48**: p. 614-616.

7.	Visher, C., N. LaVigne, and J. Travis, *Returning home: Understanding the challenges of prisoner reentry*. 2004, Urban Institute Justice Policy Center: Washington, DC. p. 1-240.

8.	Marlowe, D.B., *Evidence-based policies and practices for drug-involved offenders.* The Prison Journal, 2011. **91**(3_suppl): p. 27S-47S.

9.	Williamson, M., *Improving the health and social outcomes of people recently released from prisons in the UK*. 2006, The Sainsbury Centre for Mental Health: London, UK.

10.	Western, B., et al., *Stress and hardship after prison.* American Journal of Sociology, 2015. **120**(5): p. 1512-1547.

11.	Western, B., et al., *Study retention as bias reduction in a hard-to-reach population.* Proceedings of the National Academy of Sciences, 2016. **113**(20): p. 5477-5485.

12.	Guilcher, S.J., et al., *"Talk with me": perspectives on services for men with problem gambling and housing instability.* BMC Health Serv Res, 2016. **16**(a): p. 340.
